# Frailty trajectories and their determinants in cancer patients undergoing immunotherapy: a prospective longitudinal study

**DOI:** 10.3389/fonc.2025.1684827

**Published:** 2025-11-21

**Authors:** Wei Zhu, Juan Ji, Yiqun Yang

**Affiliations:** 1 Department of General Surgery, The First Affiliated Hospital of Soochow University, Suzhou, China; 2 Wound Care Clinic, The First Affiliated Hospital of Soochow University, Suzhou, China; 3 Department of Nursing, The Fourth Affiliated Hospital of Soochow University, Suzhou, China; 4 Department of Nursing, The First Affiliated Hospital of Soochow University, Suzhou, China

**Keywords:** cancer, immune checkpoint inhibitors, immunotherapy, frailty, trajectories, influencing factors

## Abstract

**Purpose:**

Frailty can impact the prognosis of cancer patients. We aimed to elucidate longitudinal frailty trajectories in cancer patients undergoing immunotherapy and to analyze the factors influencing these trajectories.

**Methods:**

A prospective observational cohort study was conducted among cancer patients scheduled for immunotherapy from December 2022 to November 2023. Sociodemographic and disease-related information was collected. The Chinese version of the Tilburg Frailty Indicator, the Activities of Daily Living scale, the Hospital Anxiety and Depression Scale, the Nutritional Risk Screening 2002, and the Social Support Rating Scale were used to assess patients before the first immunotherapy session (T_0_) and 1 week after each subsequent immunotherapy session until the sixth cycle (T_1_–T_6_). A growth mixed model was applied to explore frailty trajectories. Univariate and multivariate logistic regression analyses were performed to identify variables associated with each trajectory.

**Results:**

A total of 205 patients completed the treatment cycles and were included in the analysis. The overall frailty score demonstrated significant changes (p = 0.037), with an initial increase followed by a subsequent decrease during the six treatment cycles. Four distinct frailty trajectories were identified: the “persistently non-frail group” (Class 1), the “persistently frail group” (Class 2), the “frailty deterioration–remission group” (Class 3), and the “frailty remission–deterioration group” (Class 4). Compared with Class 1, patients in Class 2 were more likely to have nutritional risk (odds ratio [OR] = 4.173, 95% confidence interval [CI]: 1.637–12.664) and live in rural areas (OR = 6.869, 95% CI: 2.589–18.223), while the likelihood of being male was significantly lower (OR = 0.365, 95%, CI: 0.136–0.982). In Class 3, more patients had depression (OR = 6.663, 95% CI: 2.266–19.592), had low social support (OR = 9.483, 95% CI: 1.493–60.249), and were dependent on their spouses (OR = 5.728, 95% CI: 1.584–20.716) or their children for care (OR = 7.847, 95% CI: 1.994–30.885); however, being male and the presence of anxiety were associated with lower odds (OR = 0.316, 95% CI: 0.122–0.815; OR = 0.281, 95% CI: 0.100–0.789, respectively). Patients with distant tumor metastasis (OR = 12.712, 95% CI: 2.930–53.988), pre-treatment frailty (OR = 8.427, 95% CI: 1.973–36.003), and no history of chemotherapy (OR = 0.182, 95% CI: 0.033–0.994) were more likely to be in Class 4.

**Conclusions:**

There was significant heterogeneity in the frailty trajectories of cancer patients undergoing immunotherapy. Identifying factors associated with different frailty trajectories is crucial for implementing targeted interventions to improve prognosis in these patients.

## Introduction

According to the International Agency for Research on Cancer (1, 2), the number of new cancer cases in China was approximately 4.57 million in 2020, accounting for 23.7% of all cases worldwide. The number of cancer-related deaths was approximately 3 million, representing 30% of global cancer deaths. With an aging population, the prevalence of cancer is expected to continue increasing. The prevention and treatment of cancer have therefore become major public health concerns.

In recent years, in addition to traditional surgery, chemotherapy, and radiotherapy, immunotherapy—represented by immune checkpoint inhibitors (ICIs)—has developed rapidly. Programmed death receptor 1 (PD-1), programmed death ligand 1 (PD-L1), and cytotoxic T-lymphocyte–associated protein 4 (CTLA-4) inhibitors, which activate T cells by blocking the interaction between immune checkpoint proteins and inhibitory antibodies, thereby killing cancer cells. These agents have been widely used in patients with lung, esophageal, colorectal, gallbladder, and head and neck cancers (3). Immunotherapy has brought substantial survival benefits to patients, with reported objective remission rates reaching up to 80% (4, 5). However, similar to other treatments, immunotherapy can also result in adverse outcomes. Frailty, as one of the serious health issues in patients undergoing immunotherapy, has recently drawn considerable attention (6).

In patients with cancer, accelerated aging of the immune system and excessive release of inflammatory cytokines can lead to reduced physiological reserve (7). In addition, these patients often have abnormal nutrient metabolism (8). All of these factors can contribute to frailty. Frailty is a state of reduced physiological reserve and increased vulnerability to stressors, with an elevated risk of adverse outcomes (9). It is characterized by decreased body mass, fatigue, reduced physical activity, and depressed mood (10). The risk of frailty in patients undergoing immunotherapy is high, with reported incidence rates ranging from 27.7% to 75% (11, 12). Studies have shown that frailty not only leads to a significant reduction in patients’ physical function and daily activities but also affects their quality of life (13). In addition, frailty also prolongs the hospitalization time, increases the medical costs, and may even lead to treatment interruption or fatal immune-related adverse events (irAEs), which seriously affect patient prognosis of patients (14–16).

However, most current studies on frailty in patients receiving immunotherapy are cross-sectional (8) and therefore unable to capture the dynamic progression of frailty in individuals who may experience profound biopsychosocial changes during treatment (17). Moreover, heterogeneity in frailty change trajectories among different individuals makes it difficult to draw definitive conclusions (18). Longitudinal studies incorporating a growth mixture model (GMM) can identify and classify frailty trajectories in different patients (19, 20), thereby facilitating the early identification of those at high risk for frailty and enabling the development of targeted interventions tailored to specific trajectory patterns.

Therefore, we conducted a longitudinal study to track and examine frailty trends in cancer patients undergoing immunotherapy. Our objectives were to elucidate potential categories of frailty trajectories and to analyze the factors influencing these trajectories, to provide recommendations for the early detection of frailty and the implementation of precise interventions in this patient population.

## Methods

### Study design and participant selection

We performed a prospective observational cohort study in patients scheduled for immunotherapy at the oncology and radiotherapy departments of a tertiary care hospital, the First Affiliated Hospital of Soochow University, in Suzhou, Jiangsu, China, between December 2022 and November 2023. The study protocol was approved by the Ethics Review Committee of Soochow University (approval No. SUDA20221228H12). The researcher explained the purpose and process of the study, as well as privacy protection measures, to eligible patients. Informed consent was obtained from every study participant.

The inclusion criteria were as follows: 1) patients aged 18 years or older; 2) a diagnosis of malignant tumors based on pathologic reports; 3) hospitalization for first-time immunotherapy; 4) normal cognitive function and communication skills; and. 5) patients scheduled for immunotherapy alone or in combination with chemotherapy, radiotherapy, or antiangiogenic therapy, as well as those scheduled for dual immune checkpoint therapy. Patients were excluded if they had 1) severe mental illness or 2) complications, including serious physical illnesses or organ dysfunction. In addition, the following patients were excluded from the final study analysis: 1) those who died, suffered serious complications, or became sicker and were unable to remain in the study; 2) those whose treatment changed or who were transferred to another hospital; or 3) those who did not complete the treatment cycle, withdrew, or were lost to follow-up during the study period.

### Sample size calculation

According to the principle of sample size estimation for multifactor analysis, the sample size is usually at least 10–15 times the number of independent variables. In this study, the number of independent variables was 20. Therefore, we targeted a sample size of 20 variables × 10 = 200 cases. In addition, based on the Bayesian information criterion (BIC) as a fitness indicator, the sample size should be at least 200. Considering a 20% loss during the follow-up period, the final sample size was determined to be 240 cases.

### Study protocol

Sociodemographic information (sex, age, marital status, education level, place of residence, and primary caregiver) and disease-related information (tumor site, disease duration, distant metastasis status, treatment plan, and type of medication) were recorded.

Immunotherapy was administered every 3 weeks (21 days) and continued until disease progression or the development of severely intolerable immune-related adverse events, with a maximum duration of up to 2 years. In this study, the time points for baseline investigation and follow-up were established based on the characteristics of immunotherapy. These time points were before the first use of immune checkpoint inhibitors (T_0_) and 1 week after each immunotherapy session until the sixth cycle (T_1_–T_6_). Immunotherapy patients are usually discharged within 3 days; therefore, face-to-face interviews were conducted at T_0_, and telephone interviews were performed at T_1_–T_6_. At each time point from T_0_ to T_6_, we collected the following information.

### Tilburg frailty indicator

The TFI was proposed by Gobbens et al. in 2010 (17). The scale covers three dimensions—physical, psychological, and social frailty—with a total of 15 items and a score ranging from 0 to 15. A score of 5 or more indicates frailty, with higher scores reflecting greater severity. The TFI was translated into Chinese by Xi et al. (21), with a Cronbach’s α of 0.686. In this study, the Cronbach’s α was 0.638.

### Nutritional risk screening 2002

The NRS 2002 is used to assess nutritional risk and has been validated as a nutritional screening tool in oncology patients (22). It evaluates nutritional status, including body mass index (BMI), recent weight change, and dietary intake, as well as disease severity and age. The total score ranges from 0 to 7, with a score of 3 or more indicating nutritional risk.

### Social support rating scale

The SSRS was developed by Xiao et al. in 1994 to assess the level of social support (23). It consists of 10 items in three dimensions: objective support (3 items), subjective support (4 items), and utilization of social support (3 items). Higher total scores indicate greater social support. Scores of ≤22, 23–44, and ≥45 correspond to low, medium, and high levels of social support, respectively. In this study, the Cronbach’s α was 0.834.

### Hospital anxiety and depression scale

The HADS was developed by Zigmond et al. to assess psychological distress in individuals (24). The scale consists of two subscales, anxiety (HADS-A) and depression (HADS-D), with each subscale containing seven items. Each item is scored from 0 to 3, and each subscale score ranges from 0 to 21. A HADS-A score of ≥8 or a HADS-D score of ≥8 indicates significant symptoms. In this study, the Cronbach’s α was 0.942.

### Activity of daily living scale

The ADL scale was developed by Lawton and Brody in 1969 and consists of two parts: physical self-care and instrumental activities of daily living, with a total of 14 items (25). The total score ranges from 14 to 56. Scores of 14, 15–21, and ≥22 are considered normal, mild to moderate impairment, and severe impairment, respectively. In this study, the Cronbach’s α was 0.926.

### Statistical analysis

Data analysis was conducted using SPSS 26.0 (IBM, USA) and Mplus 8.3 (Muthén & Muthén, USA). Continuous variables were presented as mean ± standard deviation or median with interquartile range (IQR) and compared using the Student’s *t*-test or Mann–Whitney *U*-test, depending on the results of the normality test results. Categorical variables were presented as numbers and percentages and compared using the chi-square test or Fisher’s exact test. Differences between patients who completed the study and those lost to follow-up were analyzed. Repeated-measures analysis of variance (ANOVA) was used to investigate longitudinal changes from T_0_ to T_6_.

The growth mixture model (GMM), which can identify subgroups within heterogeneous populations, was used to explore frailty trajectories. The model fit indices for frailty trajectories included the Akaike information criterion (AIC), Bayesian information criterion (BIC), sample size–adjusted BIC (aBIC), entropy, Lo–Mendell–Rubin likelihood ratio test (LRT), and bootstrap-based likelihood ratio test (BLRT). The best model was determined by combining practical significance with fit indices. Factors influencing the frailty trajectory were first analyzed using univariate analysis. Variables with *p* ≤ 0.2 in the univariate analysis were subsequently included in a multivariate logistic regression analysis. A *p* < 0.05 was considered statistically significant.

## Results

### Patient characteristics

A total of 241 patients were enrolled at T_0_, with 36 patients dropping out during the study period due to serious illness, death, or changes in the treatment regimen. Ultimately, 205 patients completed the survey at all time points (response rate: 85.1%). Their baseline characteristics are shown in **Table 1**. The patients’ ages ranged from 34 to 85 years. The majority were male (67.8%) and married (91.2%), with 39.5% having an education level of primary school or lower, 55.6% having a body mass index (BMI) between 18.5 and 23.9 kg/m², 62.4% living in urban areas, and 43.9% depending on their spouses for care. In addition, 118 patients (57.5%) had gastrointestinal tract cancer, and most (75.6%) had no distant cancer metastasis. A majority (58.1%) had a disease duration <6 months, and 60.0% and 29.8% had undergone surgery and chemotherapy, respectively, prior to immunotherapy. Immunotherapy was most commonly combined with chemotherapy (74.6%), and 93.7% of patients used PD-1 inhibitors. Overall, 23.4% were at risk of malnutrition, 44.4% experienced anxiety, 63.9% exhibited symptoms of depression, 45.4% had severe functional impairments, and 15.6% reported low levels of social support.

**Table 1:** Patient baseline characteristics (n=205).

Characteristics	Number (n)	Percentage (%)
Sex		
Female	66	32.2
Male	139	67.8
Age, year		
<65	88	42.9
65-75	97	47.3
≥75	20	9.8
Educational background		
Primary and lower	81	39.5
Junior	65	31.7
High school / technical secondary school	37	18.1
College or above	22	10.7
Marital status		
Married	187	91.2
Single	18	8.8
Body mass index, kg/m^2^		
<18.5	29	14.2
18.5-23.9	114	55.6
≥24	62	30.2
Residence		
Village	77	37.6
City	128	62.4
Caregiver		
Spouse	90	43.9
Children	70	34.1
Self	45	22.0
Tumor site		
Gastrointestinal tract	118	57.5
Lung	48	23.4
Urogenital system	9	4.4
Head and neck	20	9.8
Others	10	4.9
Duration of disease, months		
<6	119	58.1
6-12	23	11.2
12-24	23	11.2
≥24	40	19.5
Comorbidity		
0	96	46.8
1	77	37.6
2	32	15.6
Distant metastasis	50	24.4
Surgery	123	60.0
History of chemotherapy	61	29.8
Treatment regimen		
ICIs	9	4.4
ICIs+antiangiogenic	16	7.8
ICIs+chemotherapy	153	74.6
ICIs+chemotherapy+antiangiogenic	15	7.3
ICIs+chemotherapy+radiotherapy	12	5.9
ICIs		
PD-1	192	93.7
PD-L1	13	6.3
NRS2002		
Yes	48	23.4
No	157	76.6
HADS-A		
Yes	91	44.4
No	114	55.6
HADS-D		
Yes	131	63.9
No	74	36.1
ADL		
Normal	56	27.3
Mild to moderate dysfunction	56	27.3
Severe dysfunction	93	45.4
SSRS		
Low	32	15.6
Medium	150	73.2
High	23	11.2

ADL, activity of daily living; ICIs, immune checkpoint inhibitors; HADS-A, hospital anxiety and depression-anxiety; HADS-D, hospital anxiety and depression-depression; NRS 2002, nutritional risk screening 2002; PD-1, programmed death receptor 1; PD-L1, programmed death ligand 1; SSRS, social support rating scale; TFI, Tilburg frailty indicator.

### Prevalence of frailty and trajectories of frailty

During immunotherapy, the incidence of frailty in cancer patients from pre-treatment to the sixth treatment cycle (T_0_–T_6_) was 53.2%, 63.4%, 67.3%, 63.9%, 63.9%, 61.0%, and 54.1%, respectively (**Table 2**). Repeated-measures ANOVA results showed that frailty scores differed significantly across the seven time points (*p* = 0.037). Frailty scores showed an increasing trend from T_0_ to T_3_, followed by a decreasing trend to T_6_, with scores at T6 still higher than those at T_0_.

**Table 2:** Changes in the incidence and scores of frailty during immunotherapy.

Timepoint	Frailty, n (%)	Tilburg frailty indicator			
		Total score	Physical	Mental	Social
T0	109(53.2)	4.71±2.11	2.44±1.44	1.72±1.00	0.54±0.72
T1	130(63.4)	5.38±2.16	2.98±1.44	1.83±1.01	0.57±0.80
T2	138(67.3)	5.63±2.31	3.17±1.53	1.88±0.98	0.58±0.79
T3	131(63.9)	5.73±2.56	3.31±1.61	1.82±1.08	0.60±0.78
T4	131(63.9)	5.61±2.47	3.20±1.55	1.77±1.05	0.64±0.83
T5	125(61.0)	5.49±2.67	3.18±1.71	1.64±1.07	0.66±0.83
T6	111(54.1)	5.36±3.26	3.18±1.98	1.49±1.18	0.69±0.85
*F*		11.522	17.129	4.924	2.828
*P*		0.037	<0.001	<0.001	0.010

The total and each dimension scores are presented as mean ± standard deviation.

**Figure 1:**
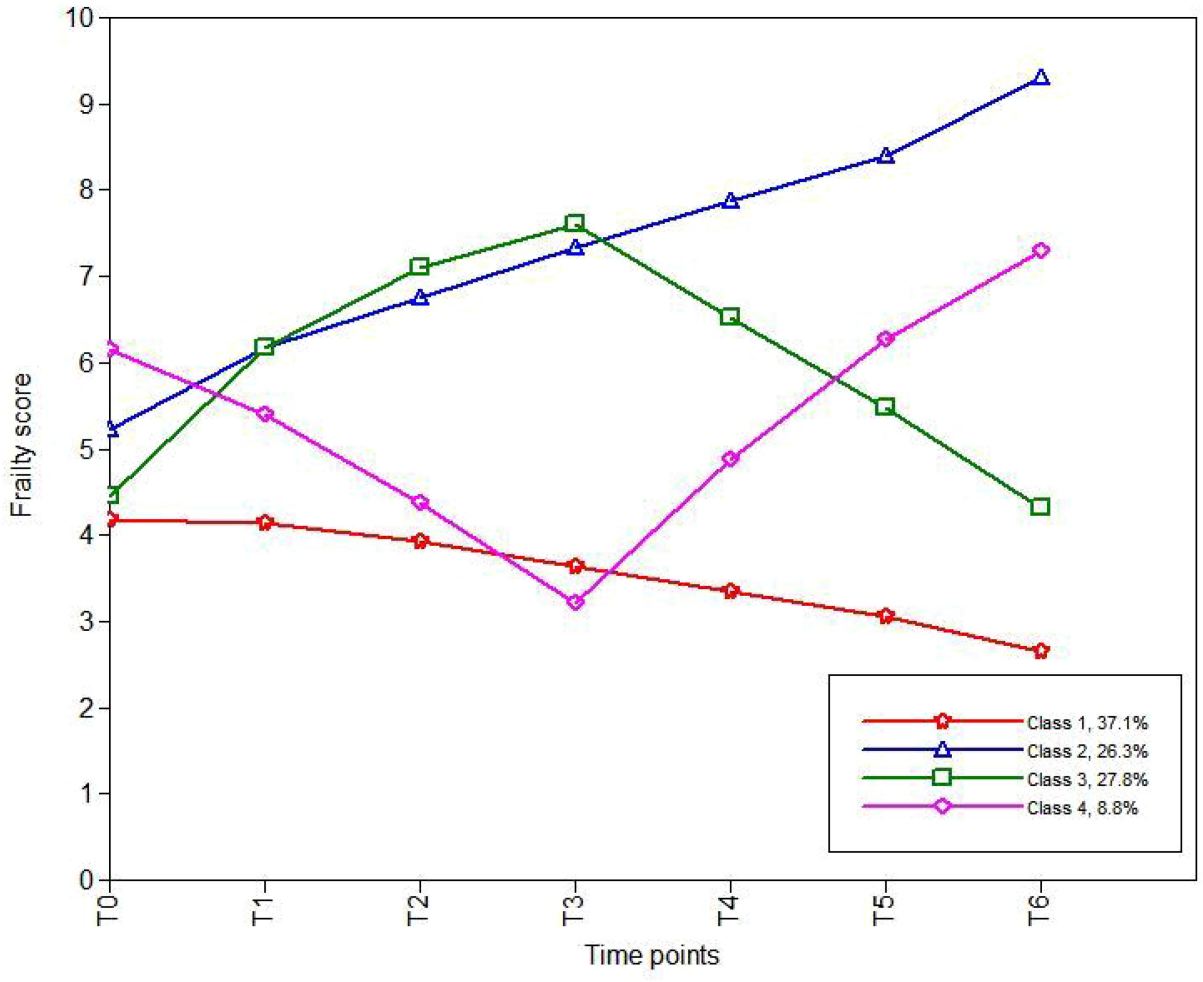
Four models of frailty trajectories in cancer patients under immunotherapy.

Based on the AIC, BIC, and aBIC values, four frailty trajectories were identified in patients receiving cancer immunotherapy (**Table 3**; **Figure 1**). They were named according to their slopes and intercepts:

- Category 1 (C1): 76 patients (37.1%). The baseline frailty score was 4.270 (standard error [SE] 0.313; P < 0.001) with a slope of -0.129 (SE 0.072; P = 0.071), indicating that patients in C1 were not frail at baseline and showed a slight downward trend. Thus, C1 was named the “persistently non-frail group”.
- Category 2 (C2): 54 patients (26.3%). Patients in C2 were initially frail (intercept: 5.565, SE 0.276; p < 0.001), and their frailty level continued to rise over the subsequent six time points (slope: 0.550, SE 0.073; P< 0.001). Therefore, C2 was named the “persistently frail group.”
- Category 3 (C3): 57 patients (27.8%). C3 was named the “frailty deterioration–remission group.” Patients in this group were non-frail at baseline but showed an upward trend (intercept: 4.804, SE 0.337; P < 0.001; slope: 1.664, SE 0.137; P < 0.001). They became extremely frail by the third cycle, after which the frailty score declined and was alleviated to a non-frail state by the sixth cycle.
- Category 4 (C4): 18 patients (8.8%). C4, named the “frailty remission– deterioration group,” included patients who were highly frail at baseline but experienced rapid remission (intercept: 6.539, SE 0.648; P < 0.001; slope: −1.657, SE 0.226; P < 0.001). However, their frailty deteriorated after the third cycle, and by the sixth cycle, the frailty severity was worse than that at baseline.

**Table 3:** Latent class analysis on the trajectory of frailty during immunotherapy.

Class	AIC	BIC	aBIC	Entropy	*P*		Class probability
					LMR	BLRT	
1	4444.719	4497.887	4447.193	–	–	–	1
2	4379.089	4445.549	4382.183	0.901	0.001	0.001	0.3605/0.6395
3	4376.990	4456.742	4380.702	0.837	0.552	0.150	0.3112/0.1659/0.5229
4	4340.559	4433.604	4344.890	0.906	0.018	0.001	0.3707/0.2634/0.2781/0.0878
5	4358.559	4454.896	4353.509	0.919	0.500	0.429	0.2769/0.0844/0.000/0.2783/0.3603

AIC, Akaike information criteria; BIC, Bayesian information criterion; aBIC, sample-size adjusted BIC; LMR, lo-mendell-rubin; BLRT, bootstrapped-likelihood ratio test.

### Univariate and multivariate analysis of the influencing factors of frailty trajectories

We further identified the influencing factors of frailty trajectories through logistic regression analysis (**Tables 4** and **5**). Compared with C1, patients in C2 (persistently frail group) were more likely to be at nutritional risk (odds ratio [OR] = 4.173; *p* = 0.004) and reside in rural areas (OR = 6.896; *p* < 0.001). In addition, the likelihood of being male in this group was significantly lower than that of being female (OR = 0.365; *p* = 0.046).

**Table 4:** Univariate analysis of variables associated with the different classes of frailty trajectories.

Variables	Class 1 (n=76)	Class 2 (n=54)	Class 3 (n=57)	Class 4 (n=18)	*χ^2^*	*P*
Sex					7.210	0.066
Female	16 (21.1)	22 (40.7)	22 (38.6)	6 (33.3)		
Male	60 (78.9)	32 (59.3)	35 (61.4)	12 (66.7)		
Age, years					4.956	0.549
<65	34 (44.7)	25 (46.3)	23 (40.4)	6 (33.3)		
65-75	38 (50.0)	21 (38.9)	28 (49.1)	10 (55.6)		
≥75	4 (5.3)	8 (14.8)	6 (10.5)	2 (11.1)		
Educational background					19.785	0.019
Primary and lower	20 (26.3)	29 (53.7)	26 (45.6)	6 (33.3)		
Junior	33 (43.4)	12 (22.2)	15 (26.3)	5 (27.8)		
High school / technical secondary school	16 (21.1)	10 (18.5)	9 (15.8)	2 (11.1)		
College or above	7 (9.2)	3 (5.6)	7 (12.3)	5 (27.8)		
Marital status					1.056	0.825
Married	70 (92.1)	48 (88.9)	53 (93.0)	16 (88.9)		
Single	6 (7.9)	6 (11.1)	4 (7.0)	2 (11.1)		
Body mass index, kg/m^2^					3.816	0.702
<18.5	11 (14.5)	5 (9.3)	9 (15.8)	4 (22.2)		
18.5~23.9	39 (51.3)	35 (64.8)	31 (54.4)	9 (50.0)		
≥24	26 (34.2)	14 (25.9)	17 (29.8)	5 (27.8)		
Residence					15.204	0.002
Village	21 (27.6)	32 (59.3)	19 (33.3)	5 (27.8)		
City	55 (72.4)	22 (40.7)	38 (66.7)	13 (72.2)		
Caregiver					15.807	0.015
Spouse	34 (44.7)	20 (37.0)	26 (45.6)	10 (55.6)		
Children	19 (25.0)	25 (46.3)	24 (42.1)	2 (11.1)		
Self	23 (30.3)	9 (16.7)	7 (12.3)	6 (33.3)		
Tumor location					11.309	0.457
Gastrointestinal tract	43 (56.6)	35 (64.8)	29 (50.9)	11 (61.1)		
Lung	21 (27.6)	10 (18.5)	15 (26.3)	2 (11.1)		
Urogenital system	2 (2.6)	2 (3.7)	2 (3.5)	3 (16.7)		
Head and neck	8 (10.5)	5 (9.3)	6 (10.5)	1 (5.6)		
Others	2 (2.6)	2 (3.7)	5 (8.8)	1 (5.6)		
Duration of disease, months					7.372	0.598
<6	41 (53.9)	34 (63.0)	37 (64.9)	7 (38.9)		
6-12	8 (10.5)	4 (7.4)	8 (14.0)	3 (16.7)		
12-24	10 (13.2)	5 (9.3)	5 (8.8)	3 (16.7)		
≥24	17 (22.4)	11 (20.4)	7 (12.3)	5 (27.8)		
Treatment regimen					8.206	0.753
ICIs	4 (5.3)	2 (3.7)	2 (3.5)	1 (5.6)		
ICIs+antiangiogenic	5 (6.6)	4 (7.4)	4 (7.0)	3 (16.7)		
ICIs+chemotherapy	55 (72.4)	43 (79.6)	44 (77.2)	11 (61.1)		
ICIs+chemotherapy+antiangiogenic	7 (9.2)	4 (7.4)	2 (3.5)	2 (11.1)		
ICIs+chemotherapy+radiotherapy	5 (6.6)	1 (1.9)	5 (8.8)	1 (5.6)		
ICIs					5.723	0.096
PD-1	74 (97.4)	49 (90.7)	54 (94.7)	15 (83.3)		
PD-L1	2 (2.6)	5 (9.3)	3 (5.3)	3 (16.7)		
Distant metastasis	13 (17.1)	19 (35.2)	8 (14.0)	10 (55.6)	18.394	<0.001
Surgery	40 (52.6)	36 (66.7)	37 (64.9)	10 (55.6)	3.441	0.329
History of chemotherapy	23 (30.3)	22 (40.7)	13 (22.8)	3 (16.7)	5.919	0.116
Comorbidity					6.810	0.339
0	33 (43.4)	25 (46.3)	30 (52.6)	8 (44.4)		
1	25 (32.9)	22 (40.7)	22 (38.6)	8 (44.4)		
≥2	18 (23.7)	7 (13.0)	5 (8.8)	2 (11.1)		
Pre-immunotherapy frailty	31 (40.8)	34 (63.0)	30 (52.6)	14 (53.2)	11.142	0.011
NRS2002					12.614	0.006
Yes	12 (15.8)	22 (40.7)	10 (17.5)	4 (22.2)		
No	64 (84.2)	32 (59.3)	47 (82.5)	14 (77.8)		
HADS-A					12.913	0.005
Yes	26 (34.2)	35 (64.8)	23 (40.4)	7 (38.9)		
No	50 (65.8)	19 (35.2)	34 (59.6)	11 (61.1)		
HADS-D					19.981	<0.001
Yes	37 (48.7)	40 (74.1)	46 (80.7)	8 (44.4)		
No	39 (51.3)	14 (25.9)	11 (19.3)	10 (55.6)		
SSRS					16.271	0.012
Low	4 (5.3)	10 (18.5)	16 (28.1)	2 (11.1)		
Medium	63 (82.9)	40 (74.1)	35 (61.4)	12 (66.7)		
High	9 (11.8)	4 (7.4)	6 (10.5)	4 (22.2)		
ADL					10.376	0.110
Normal	27 (35.5)	12 (22.2)	13 (22.8)	4 (22.2)		
Mild to moderate dysfunction	23 (30.3)	10 (18.5)	16 (28.1)	7 (38.9)		
Severe dysfunction	26 (34.2)	32 (59.3)	28 (49.1)	7 (38.9)		

ADL, Activity of daily living; ICIs, immune checkpoint inhibitors; HADS-A, hospital anxiety and depression-anxiety; HADS-D, hospital anxiety and depression-depression; NRS 2002, nutritional risk screening 2002; PD-1, programmed death receptor 1; PD-L1, programmed death ligand 1; SSRS, social support rating scale.

**Table 5:** Multivariate logistic regression analysis of variables associated with the frailty trajectories.

Class	B	Standard error	Wald	*P*	Odd ratio	95% confidence interval
C2						
Male	-1.007	0.504	3.983	0.046	0.365	0.136~0.982
Nutritional risk	1.516	0.522	8.436	0.004	4.173	1.637~12.664
Village	1.927	0.498	14.987	<0.001	6.869	2.589~18.223
C3						
Male	-1.153	0.484	5.681	0.017	0.316	0.122~0.815
Anxiety	-1.268	0.526	5.814	0.016	0.281	0.100~0.789
Depression	1.897	0.550	11.876	0.001	6.663	2.266~19.592
Social support	2.250	0.943	5.686	0.017	9.483	1.493~60.249
Spouse care	1.745	0.656	7.081	0.008	5.728	1.584~20.716
Children care	2.060	0.699	8.685	0.003	7.847	1.994~30.885
C4						
Distant metastasis	2.543	0.738	11.874	0.001	12.712	2.93~53.988
Chemotherapy	-1.702	0.865	3.870	0.049	0.182	0.033~0.994
Pre-immunotherapy frailty	2.131	0.741	8.276	0.004	8.427	1.973~36.003

For C3 (frailty deterioration–remission group), the influencing factors included presence of depression (OR = 6.663; *p* < 0.001), lower levels of social support (OR = 9.483; *p* = 0.017), and requiring care from their spouses or children (OR = 5.728; *p* = 0.008; OR = 7.847; *p* = 0.003, respectively). However, being male and presence of anxiety were associated with lower odds (OR = 0.316; *p* = 0.017; OR = 0.281; *p* = 0.016, respectively).Patients with distant tumor metastasis (OR = 12.712; *p* = 0.001), pre-treatment frailty (OR = 8.427; *p* = 0.004), and no history of chemotherapy (OR = 0.182; *p* = 0.049) were more likely to be in C4 (frailty remission–deterioration group).

## Discussion

We investigated the frailty status and trajectories of cancer patients undergoing immunotherapy and found a relatively high incidence of frailty during treatment. The pre-treatment frailty prevalence was 53.2%, peaking at 67.3% during therapy. Compared with other therapeutic modalities, the frailty incidence observed in this study was slightly higher. Bruijnen et al. found that the incidence of frailty among 98 melanoma patients treated with PD-1 inhibitors was 29% (15). By contrast, Gomes et al. reported a frailty incidence of around 50% among elderly cancer patients undergoing immunotherapy (14). The reason for this discrepancy might be due to differences in study populations and assessment scales. Our study included patients with multiple tumor types and primarily utilized the TFI for frailty assessment. The TFI is designed to measure three dimensions—physical, psychological, and social frailty—and has strong potential to identify frail patients. During immunotherapy, cancer patients often experience reduced comfort levels and nutritional disturbances (26), attributable not only to tumor-related cachexia but also to immune-related adverse events. In addition, these patients frequently face negative emotional states such as anxiety and diminished social engagement (27, 28), leading to multidimensional impacts on physical, psychological, and social status. Therefore, immunotherapy patients exhibit greater heterogeneity in frailty trajectories. A longitudinal study on breast cancer patients has reported three trajectories of frailty: “remained robust,” “started and remained pre-frail,” and “initially had nearly frail scores and became more frail” (29). Miao et al. followed 381 gastric cancer patients (aged ≥60 years) undergoing radical gastrectomy and identified three trajectory patterns: “frailty remission,” “persistently frail,” and “frailty progression” (18). Although these studies have identified three types of frailty trajectories, most of them only reported only two trends—progression or remission. In contrast, we have identified more pronounced fluctuations in frailty trajectories, which might be due to interindividual variability in immune responses, adaptive capacity, and recovery potential. Miao et al. also found that the “persistently frail” and “frailty progression” trajectories were significantly associated with worse outcomes (18). However, the relationship between frailty trajectories and clinical outcomes in immunotherapy patients remains to be further investigated. Future studies should validate this association to facilitate timely frailty management in this patient population.

Sex, residence, anxiety and depression, prior chemotherapy history, nutritional risk, social support, distant tumor metastasis, and baseline frailty status were key contributors to heterogeneity in frailty trajectories among cancer patients undergoing immunotherapy, consistent with existing research. Studies have indicated that females aged 45–79 years have a higher risk of frailty (30), potentially due to aging, abdominal obesity, and decreased estrogen levels, which predispose them to greater comorbidity burdens and sarcopenia. Xin et al. reported that rural residents were more vulnerable to frailty among Chinese older adults (31). This disparity may stem from limited healthcare resources, insufficient health literacy, and heightened physical and psychological stress associated with long-distance medical access.

Patients reliant on familial care exhibited exacerbated frailty severity, perpetuating a vicious cycle of declining functional autonomy. Metastatic disease worsens frailty due to progressive deterioration of physical function, often surpassing baseline frailty levels (32). Contrary to previous studies identifying chemotherapy history as a risk factor for symptom burden in immunotherapy patients (33), our findings suggested a protective trend. This discrepancy may arise from adaptive tolerance developed during prior chemotherapy-induced symptom burden, enabling better resilience to immunotherapy-related stressors. Further investigations are warranted to clarify the temporal relationship between chemotherapy exposure and frailty progression in the context of immunotherapy.

Notably, anxiety emerged as a protective factor in our cohort, whereas depression increased frailty risk. Potential mechanisms could include anxiety-driven proactive healthcare-seeking behaviors and improved self-management, which may mitigate prolonged psychological inertia. In parallel, robust economic and emotional support from social networks may significantly alleviate negative emotional states (34). Patients with nutritional risk demonstrated progressive frailty escalation due to impaired nutrient absorption and accelerated muscle catabolism. Pre-existing frailty at treatment initiation predicted poorer stress resilience, highlighting the need for targeted preventive strategies that address baseline frailty.

This study aimed to investigate the developmental trends and trajectories of frailty in cancer patients undergoing immunotherapy. Its strengths include a longitudinal study design, the use of a multidimensional frailty assessment scale encompassing physiological, psychological, and social domains, and follow-up monitoring over six treatment cycles. Additionally, heterogeneous frailty trajectories were identified through mixed-effects modeling.

However, this study had several limitations. First, due to serious illness, death, or changes in treatment, we excluded 36 patients who did not complete all six cycles of treatment cycles. This might have excluded the weakest patients. Furthermore, the observation period was confined to six immunotherapy cycles, whereas immunotherapy can be a long-term therapeutic process. Future research should prioritize extended longitudinal studies to elucidate the dynamic evolution of frailty and its relationship with clinical outcomes in immunotherapy patients. Multicenter investigations are also required to improve the generalizability of the study results.

## Conclusion

Patients undergoing immune checkpoint inhibitor therapy exhibited a progressively increasing trend in frailty. Four distinct frailty developmental trajectories were identified: “persistent non-frailty,” “persistent frailty,” “frailty exacerbation–remission,” and “frailty remission–exacerbation.” These trajectories demonstrated considerable heterogeneity in both frailty status and its progression, with different contributing factors across groups. Future interventions should be tailored to the specific characteristics of each frailty trajectory to optimize patient outcomes and quality of life.

## Data Availability

The raw data supporting the conclusions of this article will be made available by the authors, without undue reservation.

## References

[1] Sung H Ferlay J Siegel RL Laversanne M Soerjomataram I Jemal A . Global cancer statistics 2020: GLOBOCAN estimates of incidence and mortality worldwide for 36 cancers in 185 countries. CA Cancer J Clin. (2021) 71:209–49. doi: 10.3322/caac.21660, PMID: 33538338

[2] Cao W Chen HD Yu YW Li N Chen WQ . Changing profiles of cancer burden worldwide and in China: a secondary analysis of the global cancer statistics 2020. Chin Med J (Engl). (2021) 134:783–91. doi: 10.1097/CM9.0000000000001474, PMID: 33734139 PMC8104205

[3] Vaddepally RK Kharel P Pandey R Garje R Chandra AB . Review of indications of FDA-approved immune checkpoint inhibitors per NCCN guidelines with the level of evidence. Cancers (Basel). (2020) 12:738. doi: 10.3390/cancers12030738, PMID: 32245016 PMC7140028

[4] Brahmer JR Rodriguez-Abreu D Robinson AG Hui R Csőszi T Fülöp A . KEYNOTE-024 5-year OS update: First-line pembrolizumab vs platinum-based chemotherapy in patients with metastatic NSCLC and PD-L1 tumour proportion score (TPS) 50%. Ann Oncol. (2020) 31:S1181–2. doi: 10.1016/j.annonc.2020.08.2284

[5] Lee CK Rha SY Kim HS Jung M Kang B Che J . A single arm phase Ib/II trial of first-line pembrolizumab, trastuzumab and chemotherapy for advanced HER2-positive gastric cancer. Nat Commun. (2022) 13:6002. doi: 10.1038/s41467-022-33267-z, PMID: 36224176 PMC9556512

[6] Muhandiramge J Orchard S Haydon A Zalcberg J . The acceleration of ageing in older patients with cancer. J Geriatr Oncol. (2021) 12:343–51. doi: 10.1016/j.jgo.2020.09.010, PMID: 32933870

[7] Elias R Hartshorn K Rahma O Lin N Snyder-Cappione JE . Aging, immune senescence, and immunotherapy: A comprehensive review. Semin Oncol. (2018) 45:187–200. doi: 10.1053/j.seminoncol.2018.08.006, PMID: 30539714

[8] Ma N Teng Y Yan L Hou Y Kan Z . Analysis of the frailty status and influencing factors of lung cancer patients treated with PD-1/PDL-1 therapy. Tianjin Nurs. (2023) 31:524–8. doi: 10.3969/j.issn.1006-9143.2023.05.005

[9] Cohen CI Benyaminov R Rahman M Ngu D Reinhardt M . Frailty: A multidimensional biopsychosocial syndrome. Med Clin North Am. (2023) 107:183–97. doi: 10.1016/j.mcna.2022.04.006, PMID: 36402498

[10] Fried LP Tangen CM Walston J Newman AB Hirsch C Gottdiener J . Frailty in older adults: evidence for a phenotype. J Gerontol A Biol Sci Med Sci. (2001) 56:M146–56. doi: 10.1093/gerona/56.3.M146, PMID: 11253156

[11] Olsson Ladjevardi C Koliadi A Rydén V Inan El-Naggar A Digkas E Valachis A . Predicting immune-related adverse events using a simplified frailty score in cancer patients treated with checkpoint inhibitors: A retrospective cohort study. Cancer Med. (2023) 12:13217–24. doi: 10.1002/cam4.6013, PMID: 37132258 PMC10315811

[12] Li J Zhang X Zhou S Zhou Y Liu X . Association between PD-1 inhibitor-related adverse events and frailty assessed by frailty index in lung cancer patients. Cancer Med. (2023) 12:9272–81. doi: 10.1002/cam4.5669, PMID: 36727563 PMC10166957

[13] Thomas CM Sklar MC Su J Xu W DeAlmeida JR Alibhai SMH . Longitudinal assessment of frailty and quality of life in patients undergoing head and neck surgery. Laryngoscope. (2021) 131:E2232–42. doi: 10.1002/lary.29375, PMID: 33427307

[14] Gomes F Lorigan P Woolley S Foden P Burns K Yorke J . A prospective cohort study on the safety of checkpoint inhibitors in older cancer patients-the ELDERS study. ESMO Open. (2021) 6:100042. doi: 10.1016/j.esmoop.2020.100042, PMID: 33516147 PMC7844568

[15] Bruijnen CP Koldenhof JJ Verheijden RJ vandenBos F Emmelot-Vonk MH Witteveen PO . Frailty and checkpoint inhibitor toxicity in older patients with melanoma. Cancer. (2022) 128:2746–52. doi: 10.1002/cncr.34230, PMID: 35439334 PMC9325486

[16] Sakakida T Ishikawa T Uchino J Tabuchi Y Komori S Asai J . Safety and tolerability of PD-1/PD-L1 inhibitors in elderly and frail patients with advanced Malignancies. Oncol Lett. (2020) 20:14. doi: 10.3892/ol.2020.11875, PMID: 32774487 PMC7406883

[17] Kim DH Rockwood K . Frailty in older adults. N Engl J Med. (2024) 391:538–48. doi: 10.1056/NEJMra2301292, PMID: 39115063 PMC11634188

[18] Miao X Guo Y Chen Y Xu X Ding L Hu J . Exploration of frailty trajectories and their associations with health outcomes in older gastric cancer survivors undergoing radical gastrectomy: A prospective longitudinal observation study. Eur J Surg Oncol. (2024) 50:107934. doi: 10.1016/j.ejso.2023.107934, PMID: 38160495

[19] Du J Zhang M Zeng J Han J Duan T Song Q . Frailty trajectories and determinants in Chinese older adults: A longitudinal study. Geriatr Nurs. (2024) 59:131–8. doi: 10.1016/j.gerinurse.2024.06.015, PMID: 39002503

[20] Jenkins ND Hoogendijk EO Armstrong JJ Lewis NA Ranson JM Rijnhart JJM . Trajectories of frailty with aging: coordinated analysis of five longitudinal studies. Innov Aging. (2022) 6:igab059. doi: 10.1093/geroni/igab059, PMID: 35233470 PMC8882228

[21] Xi X Guo G Sun J . Reliability and validity study of the chinese version of the tilburg frailty assessment scale. J Nurs. (2013) 16:1–4. doi: 10.3969/j.issn.1008-9969.2013.16.001

[22] Zhang Z Wan Z Zhu Y Zhang L Zhang L Wan H . Prevalence of malnutrition comparing NRS2002, MUST, and PG-SGA with the GLIM criteria in adults with cancer: A multi-center study. Nutrition. (2021) 83:111072. doi: 10.1016/j.nut.2020.111072, PMID: 33360034

[23] Xiao SY . Theoretical basis and research application of Social Support Rating Scale. J Clin Psychiatry. (1994) 2:98–100.

[24] Zigmond AS Snaith RP . The hospital anxiety and depression scale. Acta Psychiatr Scand. (1983) 67:361–70. doi: 10.1111/j.1600-0447.1983.tb09716.x, PMID: 6880820

[25] Lawton MP Brody EM . Assessment of older people: self-maintaining and instrumental activities of daily living. Gerontologist. (1969) 9:179–86. doi: 10.1093/geront/9.3_Part_1.179, PMID: 5349366

[26] Pansarasa O Pistono C Davin A Bordoni M Mimmi MC Guaita A . Altered immune system in frailty: Genetics and diet may influence inflammation. Ageing Res Rev. (2019) 54:100935. doi: 10.1016/j.arr.2019.100935, PMID: 31326616

[27] Guo Y Miao X Jiang X Xu T Xu Qin . Meta-analysis of factors influencing frailty in cancer patients. Chin Gen Pract. (2023) 26:989–96. doi: 10.12114/j.issn.1007-9572.2022.0773

[28] Katayama O Lee S Bae S Makino K Chiba I Harada K . The association between social activity and physical frailty among community-dwelling older adults in Japan. BMC Geriatr. (2022) 22:870. doi: 10.1186/s12877-022-03563-w, PMID: 36384448 PMC9670639

[29] Mandelblatt JS Zhou X Small BJ Ahn J Zhai W Ahles T . Deficit accumulation frailty trajectories of older breast cancer survivors and non-cancer controls: the thinking and living with cancer study. J Natl Cancer Ins. (2021) 113:1053–64. doi: 10.1093/jnci/djab003, PMID: 33484565 PMC8328973

[30] Fan J Yu C Guo Y Bian Z Sun Z Yang L . Frailty index and all-cause and cause-specific mortality in Chinese adults: a prospective cohort study. Lancet Public Health. (2020) 5:e650–60. doi: 10.1016/S2468-2667(20)30113-4, PMID: 33271078 PMC7708389

[31] Qi X Li Y Hu J Meng L Zeng P Shi J . Prevalence of social frailty and its associated factors in the older Chinese population: a national cross-sectional study. BMC Geriatr. (2023) 23:532. doi: 10.1186/s12877-023-04241-1, PMID: 37658332 PMC10474699

[32] Chen F Yi P Mao N Luo J Cai D . Analysis of the current situation and influencing factors of frailty in elderly patients with lung cancer. J Nurs. (2020) 27:7–11. doi: 10.16460/j.issn1008-9969.2020.15.007

[33] Feng LN He J Feng LX Li Y Li J Chen C . Symptoms, symptom clusters and associated factors among cancer patients receiving immune checkpoint inhibitor therapy: A cross-sectional survey. Eur J Oncol Nurs. (2023) 63:102288. doi: 10.1016/j.ejon.2023.102288, PMID: 36893574

[34] Carrasco-Díaz B Gallardo-Peralta LP Araya AX Herrera MS Pedrero V SequeiraDaza D . Physical frailty in Chilean older persons: The role of social relationships, multimorbidity, and mental health. Geriatr Nurs. (2025) 62:136–43. doi: 10.1016/j.gerinurse.2025.01.040, PMID: 39921998

